# Four-day versus five-day blood culture incubation: a prospective study of diagnostic performance using the bioMérieux BACT/ALERT VIRTUO system

**DOI:** 10.1186/s12879-026-13933-z

**Published:** 2026-07-07

**Authors:** Kerri Tron, Caroline Cullerton, Jamie Lupton, Patrick Mpwilu, Julie Samuel, Daniel Weiand

**Affiliations:** Department of Microbiology, Newcastle Upon Tyne Hospitals NHS Foundation Trust, Newcastle Upon Tyne, UK

**Keywords:** Bacteraemia, Blood culture incubation time, Bloodstream infection, Diagnostic stewardship, Laboratory workflow optimisation, Automated blood culture systems, Blood culture contamination, Antimicrobial stewardship

## Abstract

**Background:**

Blood stream infections (BSIs) are associated with sepsis, high morbidity and mortality rates, and substantial financial costs. Blood cultures are the gold standard diagnostic test for BSI, with UK Standards for Microbiology Investigations (SMI) recommending routine incubation for 5 days. However, reducing the incubation time to 4 days has been reported to adequately detect true bacteraemia whilst reducing contaminant recovery. In January 2025, Newcastle upon Tyne Hospitals implemented the bioMérieux BACT/ALERT VIRTUO system. This study compared 5-day versus 4-day blood culture incubation, assessing whether a 4-day incubation period is likely to be sufficient for detecting clinically significant bloodstream infections in a UK tertiary centre.

**Methods:**

All blood cultures processed between 22 January 2025 and 31 March 2025, inclusive, were analysed. A case series review assessed the clinical significance of all growth from blood cultures after 4 days’ incubation (> 96 h). Two doctors independently appraised the medical records to evaluate the degree to which results influenced clinical management, with double coding undertaken without inter-coder conferral. A resource impact model estimated the local operational cost of extending incubation beyond 4 days.

**Results:**

In total, 6994 blood culture sets, including 13,103 bottles, were processed from 3221 distinct patients. Only twenty-three (0.18%) bottles flagged positive beyond 4 days’ incubation, of which sixteen (69.6%) led to the isolation of contaminants and seven (30.4%) led to the isolation of clinically significant organisms. Retrospective review revealed that growth from only a single bottle yielded information that influenced a change in patient management, translating to one additional clinically meaningful result from over 13,000 extended incubations (0.008%). On the basis of the resource impact model, routine incubation of blood culture for 5 days is estimated to consume an additional 337 h of staff time annually.

**Conclusions:**

Routine incubation of blood cultures beyond 4 days is resource intensive but offers negligible clinical benefits. Our results support review of recommendations outlined in the UK SMI on Sepsis and Systemic or Disseminated infections (S 12). For laboratories equipped with automated continuous monitoring blood culture systems, a routine 4-day incubation period is likely to suffice, with extended incubation reserved for specialist-driven clinical indications.

## Background

Blood stream infections (BSIs) are of significant public health concern given their association with sepsis, high morbidity and mortality rates, and substantial financial costs [1]. As the gold standard diagnostic test for BSIs, blood cultures provide critical information for pathogen identification and antimicrobial susceptibility, thereby directly influencing clinical decision-making around treatment alongside infection prevention and control measures [2, 3]. Rapid isolation of pathogenic microorganisms from blood cultures facilitates timely targeted antimicrobial therapy and has been demonstrated to reduce mortality in patients with bacteraemia [3, 4].

NHS England emphasises the importance of regular auditing of blood culture services to enhance pathway performance across pre-analytical, analytical, and post-analytical phases of specimen processing and result interpretation [5]. Each stage encompasses multiple factors that can significantly influence test accuracy, validity, and clinical relevance.

In particular, blood culture contamination presents a persistent challenge in clinical microbiology, with contaminants accounting for approximately 2–3% of all processed cultures [6]. Contaminated cultures have multiple adverse consequences, including significant diagnostic uncertainty, which may prompt initiation of inappropriate antimicrobial therapy (risking adverse drug reactions), prolong hospitalisation, and lead to ordering of unnecessary investigations, all of which contribute to increased healthcare costs [4, 7–11].

Pre-analytical factors that influence blood culture quality include fill volumes and collection technique, both of which impact on pathogen isolation rates. Evidence suggests that blood culture yield increases by approximately 3% per millilitre of blood [3].

Regarding the analytical phase of specimen processing, blood culture system selection and incubation duration are both important factors in determining blood culture quality. Firstly, automated continuous-monitoring blood culture systems have become standard practice in clinical microbiology laboratories [4]. These systems continuously monitor changes in fluorescence, which are typically driven by changes in bottle-specific gas concentrations, enabling much earlier detection of microbial growth compared with manual methods [12]. Secondly, the duration of blood culture incubation affects microbial yield [3, 5]. Current UK Standards for Microbiology Investigations (SMI) on Sepsis and systemic or disseminated infections (S 12) recommend routine incubation of blood cultures for 5–7 days, whilst acknowledging that 4 day incubation may be appropriate following local validation [3].

However, emerging international evidence suggests that routinely incubating blood cultures for only 4 days may be adequate when using fully automated, continuous-monitoring systems. Incubating bottles beyond this time often fails to provide clinically meaningful, additional information [12–14].

Indeed, shortening the routine blood culture incubation time to 4 days has been reported to reduce contaminant recovery. For example, AlMutawa et al. conducted a quality improvement project using the BD BACTEC system in Canada, analysing over 71,000 blood cultures over a one-year period. They determined that 99.2% of positive cultures were flagged within 4 days. Bottles that were flagged by the system beyond 4 days’ incubation rarely influenced clinical decision-making [12]. Similarly, Ransom et al. reviewed 158,710 blood culture bottles using the VIRTUO system in the United States, reporting that 98.7% flagged positive within 4 days [14]. Their analysis demonstrated that most fifth-day growth failed to provide additional information of clinical significance. Instead, such results could prompt the ordering of unnecessary investigations and delay timely discharge of patients from hospital. Finally, using the BD BACTEC FX system in Japan, Hashimoto et al. reported that 98.4% of positive cultures were detected within 4 days, concluding that fifth-day incubation may only be warranted in selected clinical cases [13].

In January 2025, we implemented the bioMérieux BACT/ALERT VIRTUO blood culture system, which replaced the previous bioMérieux BACT/ALERT 3D platform. This transition, combined with emerging literature supporting routine incubation of blood cultures for only 4 days, promoted a comprehensive re-evaluation of the existing blood culture pathway. The primary aim was to compare 5-day versus 4-day blood culture incubation using an automated continuous-monitoring system, and assess whether a 4-day incubation period is likely to be sufficient for detecting clinically significant bloodstream infections. To our knowledge, this represents the first UK-based study evaluating the potential impact of reducing routine blood culture incubation times using contemporary laboratory technology. Secondary aims included reporting other blood culture quality metrics, including contamination rates (prospectively determined by medical microbiology clinicians) and blood culture fill volumes.

## Methods

### Context and setting

Newcastle upon Tyne Hospitals NHS Foundation Trust (NUTH) is one of the UK’s largest teaching hospitals, managing approximately two million patient contacts annually. The Department of Microbiology and Virology at Newcastle Hospital Laboratories provides comprehensive diagnostic services for a broad range of specialist and tertiary care services, including solid organ and bone marrow transplantation, haematology and oncology, tertiary cardiology and cardiothoracic surgery, neurosciences, and vascular surgery [15].

In January 2025, we replaced our existing bioMérieux BACT/ALERT 3D blood culture system with the bioMérieux BACT/ALERT VIRTUO platform [16, 17]. The new instrument offers enhanced functionality compared with previous models, including comprehensive reporting of blood culture fill volume and time to detection (TTD) [18, 19].

### Local blood culture pathway

NHS England guidance recommends collecting two contemporaneous blood culture sets from all patients with suspected sepsis [5]. It is therefore expected that all adult blood cultures to comprise both aerobic and anaerobic bottles. Good collection technique, including filling each bottle with 8-10 ml of blood in adults [5, 20], is promoted through Trust-wide communications and automated feedback on bottle-specific fill volumes provided on all reports issued to the electronic patient record. All bottles are routinely incubated and automatically monitored for growth for a minimum of 5 days. Bottles that are flagged as ‘positive’ by the bioMérieux BACT/ALERT VIRTUO blood culture system routinely undergo Gram staining, subculture, and microbial identification using MALDI-TOF MS (Bruker).

### Prospective assignment of blood culture contamination status

Medical microbiology clinicians routinely categorise all blood culture isolates prospectively as either clinically significant pathogens or probable contaminants. This categorisation involves detailed clinical assessment of patient context, clinical details, microorganism speciation, blood culture fill volume, and time to detection.

### Data collection and analysis

We conducted a retrospective review of our Laboratory Information Management System (LIMS) to identify all blood cultures processed between 22 January 2025 and 31 March 2025. All data extraction, wrangling, and analysis was undertaken by means of a reproducible analytical pipeline (RAP), which is a set of standardised working practices that enable consistent, automated data processing [21] using reusable, version-controlled code [22, 23].

The RAP automatically and transparently transforms raw data extracted from the hospital’s Laboratory Information Management System (LIMS) using SQL [24] and R programming [25] into analysis-ready datasets through clearly documented, step-by-step processes that can be independently verified [26]. The extracted data are immediately processed, cleaned, quality-checked, and analysed using R programming [25], with the final results compiled into a standardised report using Quarto [27]. This approach was developed collaboratively by a data scientist, a consultant medical microbiologist, and a biomedical scientist.

Blood culture data management and statistical analysis were performed using R programming [25]. Data completeness and accuracy were verified through exploratory data analysis using tidyverse packages, specifically dplyr [28, 29]. Microbiological data were analysed using the AMR package [30–32]. Summary statistics were generated for key blood culture parameters, including time to detection (TTD), fill volumes, and contamination rates. Visualisations were created using ggplot2 and NHSRplotthedots packages [33, 34]. Temporal patterns in blood culture metrics were assessed using Anhøj rules to identify non-random variation in time-series data [35].

We also performed a retrospective evaluation of the clinical significance of all growth detected after 4 days’ incubation. Two infection specialist physicians independently reviewed patient medical records to determine the extent to which results affected clinical decision-making, with independent double-coding conducted without discussion between coders [36].

### Resource impact assessment

A resource impact model was developed to estimate the local operational costs of extending blood culture incubation beyond 4 days. The number of bottles flagging positive after 4 days’ incubation during the study period was extrapolated to provide annual estimates.

Staff time requirements for each positive blood culture set were estimated across three key roles: biomedical scientists (80 min), medical microbiology clinicians (45 min), and ward-based clinicians (45 min). These time estimates were derived from local expert consensus regarding typical workload requirements for each professional group.

The model integrated findings from the retrospective assessment of the clinical impact of all growth detected after 4 days’ incubation. Results are expressed as hours per year and approximate full-time equivalent weeks of work to provide context for resource allocation decisions.

## Results

### Demographics and totals

Between 22 January 2025 and 31 March 2025, inclusive, 6994 blood cultures (13103 bottles) were received from 3221 distinct patients. These patients had a mean and median age of 51.3 and 59 years, respectively (IQR 30–75 years, range 0–101 years).

### Blood culture positivity

An organism was isolated from 854 out of 6994 blood cultures (1210 bottles), equating to a blood culture positivity rate of 12.2% (Table 1).

Contaminants were isolated from 226 out of 6994 blood cultures (3.2%), and 226 out of 854 positive blood cultures (26.5%). Blood culture contaminants most commonly belonged to Species of Coagulase-negative Staphylococcus (CoNS) (Table 2). These findings mirror published patterns of first culture isolates and causes of contamination [37, 38].

**Table 1:** Breakdown of the most frequently isolated blood culture species in the study cohort. Data are presented as individual number and percentages for each species

Top species isolated from blood cultures*		
Organism	Count (n)	Percentage (%)
Coagulase-negative Staphylococcus (CoNS)	298	33.8
Escherichia coli	152	17.2
Other	131	14.9
Staphylococcus aureus	88	10.0
Klebsiella pneumoniae complex	49	5.6
Streptococcus mitis	29	3.3
Cutibacterium acnes	18	2.0
Micrococcus luteus	17	1.9
Pseudomonas aeruginosa	16	1.8
Proteus mirabilis	14	1.6
Enterobacter cloacae complex	12	1.4
Enterococcus faecalis	12	1.4
Enterococcus faecium	12	1.4
Streptococcus parasanguinis	9	1.0
Streptococcus salivarius	9	1.0
Klebsiella oxytoca	8	0.9
Streptococcus pneumoniae	8	0.9

*First isolate of every patient per episode, as per Hindler et al. (2007)

**Fig. 1:**
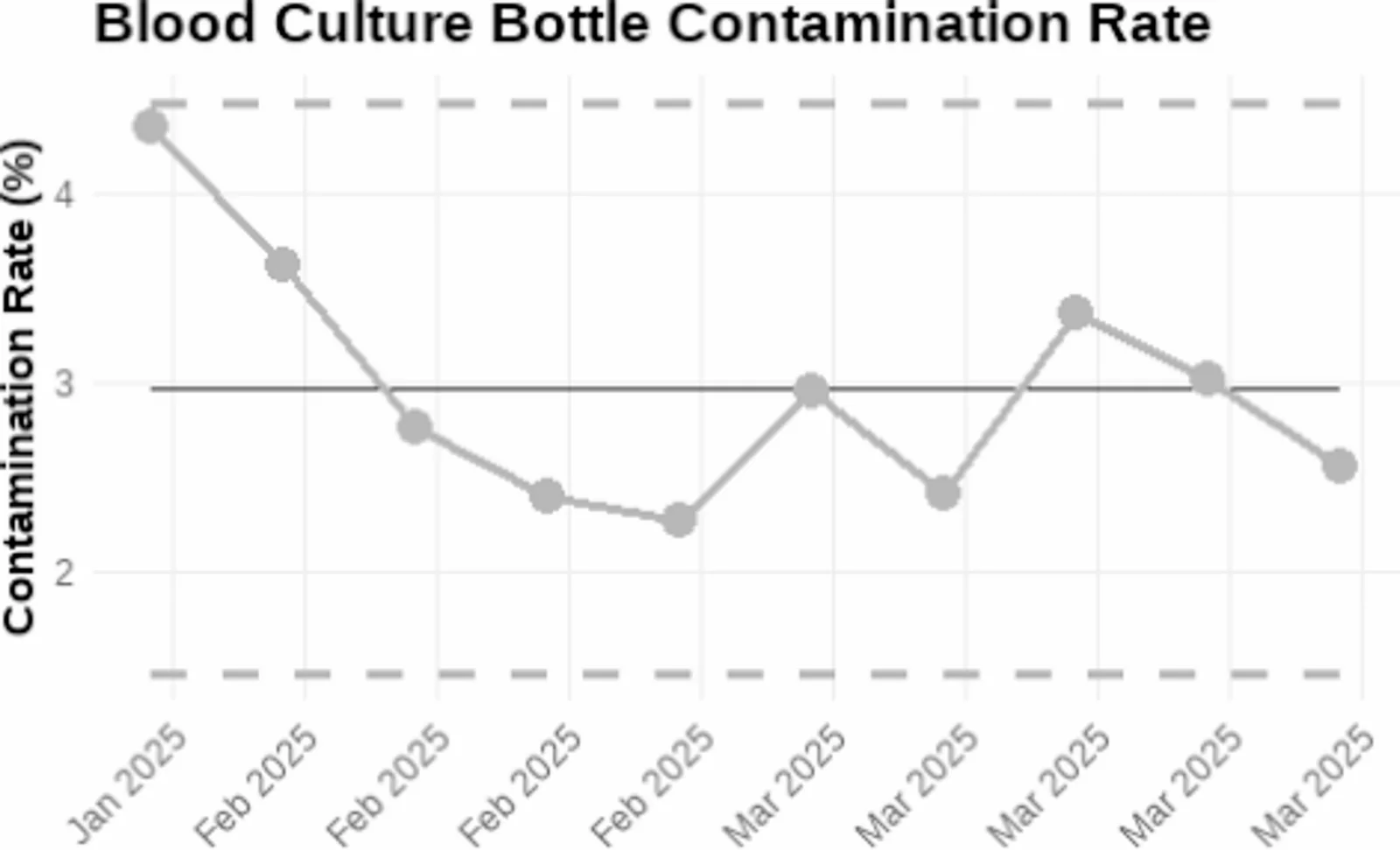
Blood culture contamination rates between January and March 2025

The blood culture contamination rate was stable throughout the entire study period, with the trend over time most likely reflecting common cause variation only [35, 39] (Fig. 1).

**Fig. 2:**
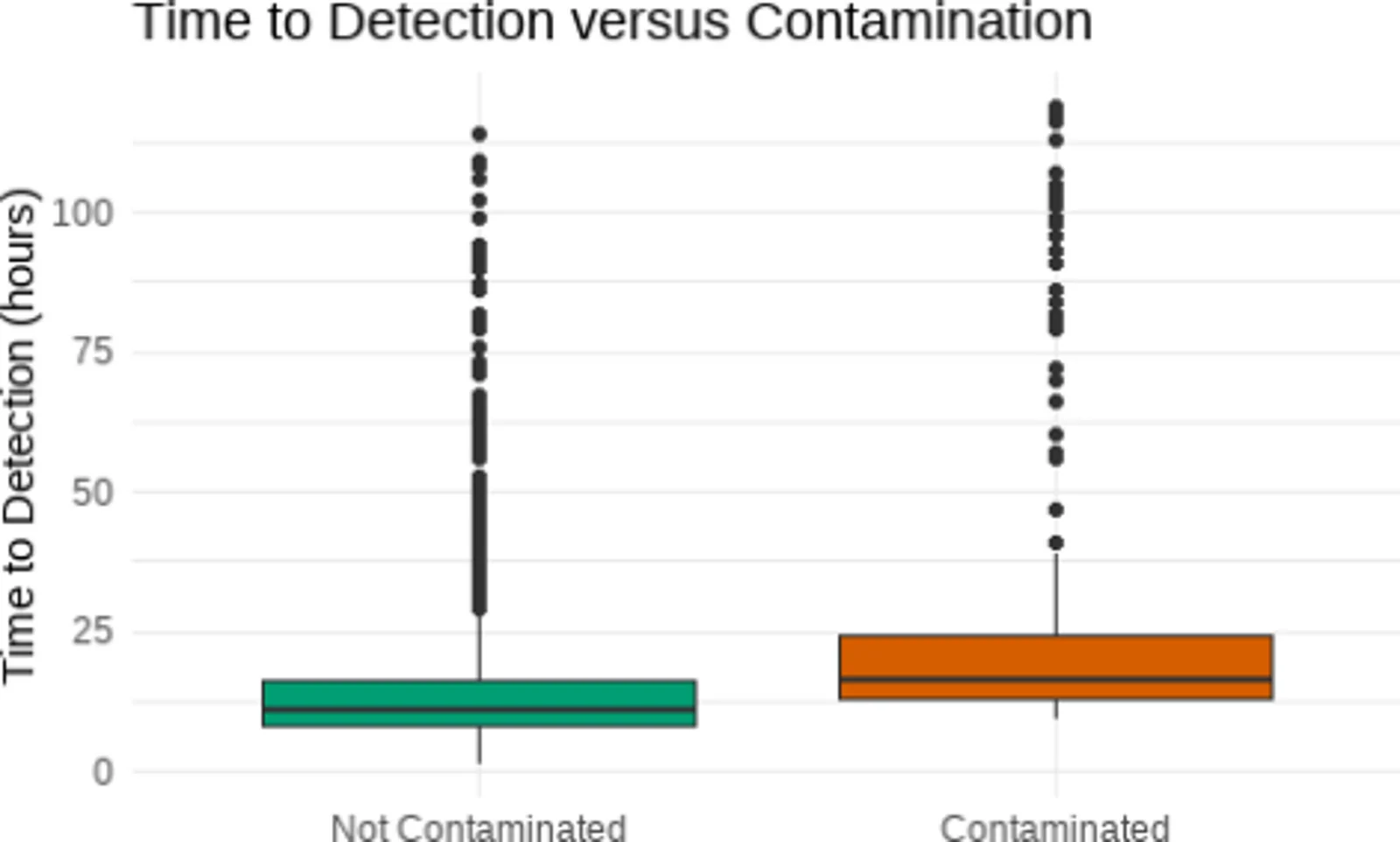
Comparison of time to detection (hours) in non-contaminated vs. contaminated blood cultures

**Table 2:** Breakdown of the most frequently isolated species in contaminated blood cultures. Data are presented as individual number and percentages for each species

Top species causing blood culture contamination*		
Organism	Count (n)	Percentage (%)
Coagulase-negative Staphylococcus (CoNS)	186	73.2
Other	17	6.7
Cutibacterium acnes	16	6.3
Micrococcus luteus	14	5.5
Streptococcus parasanguinis	4	1.6
Beta-haemolytic Streptococcus	3	1.2
Corynebacterium	2	0.8
Corynebacterium afermentans	2	0.8
Corynebacterium striatum	2	0.8
Kocuria	2	0.8
Moraxella osloensis	2	0.8
Streptococcus mitis	2	0.8
Streptococcus thermophilus	2	0.8

*First isolate of every patient per episode, as per Hindler et al. (2007)

**Table 3:** Breakdown of species isolated in the 23 blood cultures that flagged positive after 4 days’ incubation, including classification as significant growth vs contamination

Organism	Classification	Count (*n*)	Percentage (%)
Cutibacterium acnes	Contaminant	9	39.1
Gram positive Bacillus	Contaminant	5	21.7
Coagulase-negative Staphylococcus (CoNS)	Contaminant	1	4.3
Kocuria	Contaminant	1	4.3
Escherichia coli	Significant	2	8.7
Bacillus	Significant	1	4.3
Cutibacterium acnes	Significant	1	4.3
Enterobacter cloacae complex	Significant	1	4.3
Gram positive Bacillus	Significant	1	4.3
Serratia marcescens	Significant	1	4.3

### Fill volumes

Blood culture fill volume data was available for 11,664 out of 13,103 bottles (89%). The mean and median fill volumes were 7.5 ml and 8 ml, respectively (IQR 5–10 ml). Only 5996 out of 13,103 (45.8%) bottles were filled with the recommended volume of at least 8 ml blood [5, 20].

A clear relationship between fill volume and true diagnostic yield was observed. True positive yield increased from 5% in bottles containing less than 2 ml blood (*n* = 298) to 8.2% in bottles containing 8–10 ml (*n* = 2478), with a further modest increase to 8.3% in bottles containing more than 10 ml (*n* = 3412). This equates to an approximate incremental yield of 0.41% per ml of additional blood inoculated.

Among the 23 culture-positive bottles with growth after 4 days’ incubation, the mean fill volume was 8.3 ml (median 7 ml), comparable to the overall dataset mean of 7.5 ml. Notably, 69.6% of positive bottles flagging after 4 days were subsequently classified as contaminants (*n* = 16 of 23).

### Time to detection

Data on time to detection (TTD) were available for 1195 culture-positive blood culture bottles, with mean and median TTD of 19 and 13 h (IQR 9–19 h). Contaminated blood culture bottles had mean and median TTD of 27.8 and 17 h (IQR 14–28 h). In comparison, blood cultures leading to clinically significant growth had mean and median TTD of 16.4 and 12 h (IQR 8–17 h) *(*Fig. 2*)*.

Only 23 blood culture bottles were culture-positive after 4 days’ incubation. Of these, only 7 blood culture bottles (30.4%) led to the isolation of clinically significant organisms, with each positive bottle originating from a distinct patient and blood culture set. No HACEK organisms were isolated from blood cultures bottles between 4 and 5 days’ incubation. The remaining 16 out of 23 (69.6%) blood culture bottles that were culture-positive after 4 days’ incubation led to the isolation of contaminants (Table 3).

In April 2025, a retrospective case series analysis was undertaken to determine the clinical impact of all growth from blood cultures after 4 days’ incubation. This revealed that growth from only one of the 23 culture-positive bottles (4.3%) influenced patient management, whilst the remaining 22 bottles (95.7%) did not. The significant late-positive culture grew *Serratia marcescens* in a 73-year-old trauma patient with previous cultures positive for the same organism. This result prompted microbiology advice to recommence antimicrobial therapy with co-trimoxazole after a planned discontinuation of antibiotics. Overall, 13,103 bottles were set up to incubate for 5 days (120 h), of which only a single one (0.008%) yielded additional, clinically meaningful information that had a tangible impact on patient management.

### Resource impact

A resource impact model incorporating study findings estimated staff time consumed processing blood cultures incubated beyond 4 days without clinical benefit. During the 68-day study period, 23 bottles flagged positive after 4 days’ incubation, of which only one influenced patient care. Annual extrapolation suggests approximately 124 such bottles, with only 5 influencing patient care, leaving 119 bottles representing workload without patient benefit.

Processing these unnecessary positive cultures would, on the basis of local estimates, consume an estimated 159 h of biomedical scientist time, 89 h of medical microbiology clinician time, and 89 h of ward clinician time annually, totalling 337 h per year. This represents approximately 8.4 weeks of full-time equivalent work across multidisciplinary teams.

## Conclusions

The relationship between time to detection, fill volume, and contamination emphasises the importance of optimising pre-analytical quality alongside analytical performance. Our findings demonstrate that routinely extending blood culture incubation beyond 4 days yields negligible clinical benefit whilst contributing substantially to both laboratory and clinician workload.

To our knowledge, this is the first UK-based study to comprehensively evaluate the likely clinical impact of reducing routine blood culture incubation time to 4 days in a modern, tertiary care, teaching hospital setting. Our results show that incubation beyond 4 days fails to offer substantial additional, clinically impactful information. Only 0.008% of all blood cultures incubated for more than 4 days produced results that ultimately had a tangible clinical impact on patient care.

### Comparison with international evidence

Multiple international studies have shown that > 98% of positive blood cultures are detected within the first 4 days of incubation [12–14]. This conclusion is closely echoed in the results of our study, with 98.1% of positive blood culture bottles flagging within 4 days. AlMutawa et al. described the results of positive cultures post the 96 h checkpoint to be of limited clinical value, with many of these instances possessing repeat positive cultures [12]. Our case series analysis quantified that only 4.3% of blood cultures that flagged positive beyond 4 days impacted patient management, closely mirroring AlMutawa’s conclusions regarding the ineffectuality of fifth-day positive blood cultures.

### Clinical implications of late-positive cultures

In this study, only 0.18% of bottles incubated beyond 4 days were culture positive, most of which represented contamination. Growth from a single late-positive bottle (0.008%) resulted in a clinically meaningful change in patient management, with delayed detection of *Serratia marcescens* prompting recommencement of antimicrobial therapy after planned discontinuation. This highlights that, whilst occasional clinically significant late-positive cultures may still occur, routine 5-day incubation is associated with a poor diagnostic return.

Many of the organisms isolated after 4 days incubation were consistent with skin flora contamination, including Cutibacterium acnes and Gram positive bacilli [38]. Despite this, a 4 day incubation period does pose the theoretical risk of failing to identify slow-growing organisms in select clinical cases. Indeed, evaluation of blood culture incubation times by Kim et al. led to the conclusion that 5-day incubation may be required in hospitals caring for patients at increased risk for infections with slow-growing fungi or fastidious bacteria [40].

Current UK SMI S12 guidance similarly recommends consideration of extended incubation in selected cases, including suspected endocarditis, prior antimicrobial exposure, and infections caused by fungi or unusual fastidious organisms such as Brucella spp [3]. Nevertheless, evidence supporting routine prolonged incubation for HACEK organisms in modern automated continuous-monitoring blood culture systems remains limited. Analysis by Ransom et al. demonstrated HACEK organisms to flag positive within 4 days using the bioMérieux BACT/ALERT VIRTUO blood culture system, whilst review by Lamy et al. suggested that prolonged incubation periods only produce a small increased yield for some organisms, and that specialised diagnostic approaches such as serology and molecular methods may be more likely to prove diagnostic [14, 41].

Extended incubation may, however, remain clinically valuable in carefully selected circumstances. Garcia et al. demonstrated that extended blood culture incubation beyond 5 days was associated with increased identification of *Cutibacterium acnes* in patients with suspected infective endocarditis [42]. Similarly, Jeverica et al. highlighted variable and occasionally delayed recovery of *C. acnes* using automated blood culture systems in orthopaedic implant-associated infection investigations, with some systems requiring up to 14 days incubation for detection [43]. Prolonged incubation may also remain relevant in selected cases involving implantable cardiac electronic device infection, where fastidious or slow-growing organisms such as *Mycobacterium spp.*, *Nocardia spp.*, and auxotrophic staphylococci have been implicated, particularly when conventional cultures remain negative despite convincing clinical evidence of infection [44].

Consequently, any reduction in routine blood culture incubation duration should preserve flexibility for clinician- or microbiologist-directed extension in cases where infection with slow-growing, fastidious, or clinically high-risk organisms are suspected.

### Laboratory efficiency and resource optimisation

A reduction in blood culture incubation from 5 days to 4 has the potential to provide substantial laboratory efficiency gains without compromising diagnostic sensitivity. Time investment modelling estimates 337 multidisciplinary staff hours are dedicated to processing late-positive blood cultures with no overt clinical benefit on an annual basis. A 4-day incubation period could release staff capacity for tasks which enhance patient management and increase blood culture analyser availability, with only a small reduction in clinically actionable information identified after 4 days. Such improvements are particularly relevant in the context of increasing workload within NHS microbiology laboratories [45]. Furthermore, expanding blood culture analyser capacity would support the NHS England recommendation that two sets of blood cultures be taken from all patients with suspected sepsis, a standard that is currently challenging to implement due to inadequate machine availability [5].

### Contamination and pre-analytical quality

Contamination remains an important determinant of blood culture quality. Retrieval of contaminants from blood cultures can cause diagnostic uncertainty, leading to the inappropriate treatment of patients through unnecessary or unsuitable antibiotic prescriptions and prolonged hospital stays [46]. This study demonstrated a contamination rate of 3.2% which is above the largely accepted benchmark of ≤ 3%, indicating that local pre-analytical practices warrant further optimisation [47].

Time to detection (TTD) analysis demonstrated that contaminated blood culture bottles flag positive later than blood cultures leading to clinically significant growth. Furthermore, 69.6% of positive cultures post 4 days’ incubation represented contamination. These data suggest that a reduced incubation period of 4 days could work to reduce the volume of blood culture contaminants and the associated diagnostic challenges.

Regarding blood culture fill volumes, our findings reveal a compliance gap, with only 45.8% of bottles meeting the recommended threshold of at least 8 ml of blood [5, 20]. We observed a volume-dependent increase in true diagnostic yield, climbing from 5% in underfilled bottles (< 2 ml) to 8.2% in optimally filled bottles (8–10 ml). Because each additional millilitre of blood inoculated provides an incremental yield of approximately 0.41%, underfilling stands as a major driver of false-negative results. Interestingly, while underfilling severely limits initial diagnostic yield, fill volumes do not appear to dictate delayed positivity; the mean fill volume of bottles flagging after 4 days (8.3 ml) was entirely comparable to the overall dataset mean (7.5 ml).

### Implications for national guidance and practice

Current UK Standards for Microbiology Investigations (SMI S12) recommend a 5–7 day incubation period for automated blood culture systems [3]. The findings of this study, alongside consistent international evidence from contemporary continuous-monitoring systems, support re-evaluation of routine incubation duration in selected laboratory settings [12–14]. We suggest that our findings may support consideration of a routine 4-day incubation period within laboratories using automated continuous-monitoring blood culture systems similar to those evaluated in this study, provided that local validation, quality assurance, and clinical governance processes are in place.

Any reduction in routine incubation duration should preserve flexibility for extended incubation in selected clinically justified scenarios. Such cases may include suspected infective endocarditis, prior antimicrobial exposure, suspected infection with dimorphic fungi or other slow-growing or fastidious organisms such as Brucella, Cutibacterium, and nocardia. Patients with prosthetic cardiovascular material, vascular grafts, or specialist tertiary-care risk factors may particularly benefit from individualised incubation decisions directed by microbiology or infection specialists [3, 41, 48].

Consequently, decisions regarding incubation extension should consider patient context, local epidemiology, and the diagnostic capabilities of individual laboratory systems. A selective, clinician-directed approach to prolonged incubation may preserve diagnostic sensitivity in high-risk cases whilst reducing unnecessary analyser occupancy, operational costs, and contaminant recovery associated with routine 5-day incubation.

### Limitations

The retrospective nature of this study carries an inherent risk of inaccurate data entry. The LIMS data extracted consisted of automatically-assigned values, including fill volumes and time to detection (TTD), and manually entered data such as organism identification. This introduces potential for human error in both data capture and interpretation [49].

This study was conducted in a single centre which may limit the generalisability of these findings to other institutions due to variation in patient population, blood culture work flows, sepsis aetiology, and contamination profiles. Indeed, evidence from single centre trials can be insufficient in isolation to prompt practice change [50, 51].

This limitation is particularly important considering the diversity in healthcare settings and health inequalities across the UK [52]. Furthermore, this study may not be applicable to institutions that are not using the bioMérieux BACT/ALERT VIRTUO blood culture system or a similar automated blood culture system; caution is required in extrapolating results to all automated systems.

Blood culture fill volumes were not available for 1439 out of 13,103 bottles (11%). This is because the bioMérieux BACT/ALERT VIRTUO blood culture system does not report the fill volume on paediatric bottles. In addition, some bottles can return a blank or missing volume report. When this happens, it is usually caused by pre-analytical errors that disrupt the system’s optical imaging scan (for example, incorrect patient label placement).

Antimicrobial prescriptions data was not available in this study. Therefore, it was not possible to estimate the degree to which antimicrobial therapy administered prior to blood culture collection may have affected growth.

The resource impact model utilised within this study was developed using local expert consensus of multidisciplinary staff time. Whilst this model presents a useful estimate of staff time consumption, it is not validated against real-world data and assumes uniformity in operational time and workload across healthcare workers. Thus, actual time savings from a reduction in blood culture incubation periods may differ.

The sample size, while sufficient for assessing the primary outcome, may still fail to capture growth of the rarest slow-growing pathogens such as the HACEK group [14]. In addition, the relatively short study period (68 days) limits the ability to fully account for seasonal variation in bloodstream infection epidemiology and may have reduced the likelihood of capturing infrequent or slow-growing pathogens that may become apparent over longer observation periods [53].

### Future work

In order to strengthen the evidence base of this study’s recommendations, a multicentre evaluation is required. This should include institutions using a selection of different blood culture systems, serving a variety of patient populations across the UK. This would provide a more comprehensive understanding of the generalisability of these findings across settings with varying patient epidemiology, blood culture work flows, and contamination profiles.

Additionally, future research should assess the real-world implementation of a 4-day blood culture incubation period. This would allow the assessment of true time and resource savings in clinical practice, whilst also helping to elucidate the circumstances in which specialist advice would guide extended incubation. Real world implementation would offer further insight into the trade-offs between work flow efficiency and diagnostic accuracy, further helping to refine recommendations for blood culture incubation duration across varying clinical contexts.

## Data Availability

The datasets generated and analysed contain patient-level information derived from the LIMS that are subject to strict confidentiality and information governance requirements [55, 56]. Access to these data is restricted to authorised personnel. However, de-identified, aggregated summary statistics and analytical code underlying the reproducible analytical pipeline (RAP) are available upon reasonable request from the corresponding author. Researchers wishing to access aggregated data or code should contact the corresponding author with a description of their intended use and research governance approval from their own institution, to be evaluated against NHS information governance requirements and data protection regulations.

## Declarations

### **Patient and public involvement**

Patients and the public were not involved in this study design, conduct, or dissemination planning. This retrospective analysis of routinely collected laboratory data did not require patient recruitment or direct patient participation. However, the study addresses a clinically relevant question regarding optimal diagnostic testing that could impact future patient care through improved laboratory efficiency and diagnostic accuracy.

## Abbreviations

AMR: Antimicrobial resistance
BSI: Bloodstream infection
CoNS: Coagulase-negative Staphylococcus
IQR: Interquartile range
LIMS: Laboratory Information Management System
MALDI-TOF MS: Matrix-Assisted Laser Desorption/Ionisation Time-Of-Flight Mass Spectrometry
NHS: National Health Service
NUTH: Newcastle upon Tyne Hospitals NHS Foundation Trust
RAP: Reproducible analytical pipeline
SMI: Standards for microbiology Investigations
TTD: Time to detection
UK: United Kingdom
